# Factors associated with cesarean delivery in public and private hospitals in a city of northeastern Brazil: a cross-sectional study

**DOI:** 10.1186/s12884-015-0570-8

**Published:** 2015-06-05

**Authors:** Graciete Oliveira Vieira, Lorena Gabriel Fernandes, Nelson Fernandes de Oliveira, Luciana Rodrigues Silva, Tatiana de Oliveira Vieira

**Affiliations:** State University of Feira de Santana, Bahia, Brazil; Federal University of Bahia, Bahia, Brazil; Integrative Medicine Institute Teacher Fernando Figueira (IMIP), Bahia, Brazil

**Keywords:** Cesarean Section, Vaginal Birth, Heath Systems, Private Sector, Risk Factors

## Abstract

**Background:**

To evaluate the prevalence and factors associated with cesarean delivery according to whether care was provided in public or private hospitals in Brazil.

**Methods:**

This was a cross-sectional study based on a cohort of live births between April 2004 and March 2005. A total of 1,344 mother-child pairs were followed up during the first month of life. The variables analyzed were the socioeconomic and demographic characteristics of the mother and newborn, as well as the healthcare provided during pregnancy and childbirth. Hierarchical analysis was carried out for both prediction models, i.e. healthcare provision either within the Brazilian National Health System (public service) or within the private network. Prevalence and association measurement calculations were carried out. Values were considered significant when pless than or equal to 5.0 %.

**Results:**

A total of 1,019 (75,8 %) gave birth in public hospital. The prevalences of cesarean delivery were 29.9 % and 86.2 % in the public and private sectors, respectively. Through hierarchical logistic regression, the risk factors for cesarean delivery presented in the public hospital were maternal age greater than or equal to 20 years (p = 0.003), primiparity (p = 0.004), twinning (p = 0.039), prenatal care provided in the private network (p = 0.004), delivery in hospitals providing high complexity medical care (p = 0.000) and prenatal care with greater than or equal to 6 consultations (p = 0.035). In the private sector, no association was observed between the variables studied and cesarean delivery.

**Conclusions:**

The cesarean delivery rates were high in both sectors, although in the private network the rate was almost triple that of the public service. The absence of determinant factors of birth in the private sector drew attention. In planning measures against the growing cesarean rates, it is necessary to take into consideration the environmental determinants as primiparity, twinning and greater maternal age, frequent indications of primary cesarean delivery, as well as to implement actions that might improve the quality of prenatal and delivery care.

## Background

Normal/vaginal delivery respects the woman’s physiological processes and is associated with lower morbidity and mortality rates for the mother-child pair [1]. Despite the advantages of vaginal birth, there has been a progressive increase in cesarean delivery as a worldwide phenomenon since the 1960s. Currently, cesarean deliveries are the most widely performed surgical procedure among women [2]. In the United States, a country with a hegemonic healthcare model and high incorporation of technology, the incidence of cesarean delivery in 2007 was 31.8 % [2].

The World Health Organization (WHO) affirms that there is no justification for cesarean rates higher than 10 %–15 %, and defines these numbers as limiting rates [3]. Previous studies have observed two major tendencies in the distribution of surgical delivery in developing countries. Poorer countries (especially African countries) have lower rates, due to limited access to the procedure, even when indicated, with a consequent impact on morbidity-mortality among mothers and newborns. On the other hand, developing countries with a more stable economic situation, such as Latin American countries and China, have high rates [4].

Brazil has high cesarean rates. Data from 2010 show that the proportion of cesareans deliveries over the whole country was 52.3 %, with the highest rates occurring in the southern region (58.2 %) and southeastern region (58.3 %), regions in which a more medical healthcare model predominates [5]. Although the rates were also high (44.3 %) in the northeastern region, they were still lower than in the southern and southeastern regions [5]. In the state of Bahia, the prevalence was 38.4 % [5].

Although cesareans deliveries can benefit the health of the mother and child in specific situations, there needs to be an awareness of the possible risks of this procedure [6, 7]. Regarding the impacts on newborns, some studies have suggested that those born from elective cesareans deliveries present increased risk of respiratory disorders, late initiation of breastfeeding [8], and greater newborn mortality [9].

Although the clinical determinant factors for cesareans deliveries do not differ among the various regions of the world, the factors associated with increased cesarean rates are influenced by demographic and socioeconomic variables [2]. Factors relating to the healthcare model involving the medical work and preferences of the pregnant woman were important variables in the process of choosing surgical delivery [10].

In Brazil, the government has attempted administrative, managerial, educational and technical measures, without success, to control the high cesarean rates especially in the private sector, where the rates are even higher than in the public service [11]. Regional and local differences among cultural characteristics and the characteristics of the health system can influence the choice of type of delivery. For better results, it is necessary to contextualize the sociocultural determinants in addition to the current healthcare model. In this regard, the present study aimed to identify the prevalence of cesarean delivery and the associated factors, according to whether births took place in public or private hospitals, in the municipality of Feira de Santana, Bahia, Brazil.

## Methods

### Study design

This was a cross-sectional study in which data from a cohort of live births between April 2004 and March 2005 was analyzed with the aim of observing the incidence of risk factors for lactational mastitis and other outcomes over the short to medium term, in the municipality of Feira de Santana, a large-sized city at a distance of 110 km from the state capital of Bahia (Brazil) [12].

### Healthcare models for pregnant and puerperal women

At the time of this study, the prenatal healthcare network was composed of 33 primary healthcare units. There were ten healthcare units for childbirth (of which five public service). The only two neonatal intensive care units (NICUs) in the city were located in public hospitals.

Regarding obstetric care in the municipality, delivery care followed the usual Brazilian model, with two very distinct health care systems. [2] The part of the population with healthcare plans (supplementary health system), or those who pay out of pocket for health expenses, choose their doctor during the prenatal care and delivery. Meanwhile, in the public health services of the Brazilian National Health System (BNHS) there was a disconnection between the doctor who attended at prenatal consultations and the doctor present for the delivery, who was usually the obstetrician on duty. In both services, the prenatal follow-up took place in consultation offices or outpatient services, while the delivery took place in a hospital environment.

### Sample size and data collection

The present study assessed data on births at the ten hospitals that provided delivery care in the municipality during the period. The assessment was carried out by randomly choosing two hospitals every two consecutive months, with the exception of the two maternity hospitals, which were introduced separately into the cohort because they attended greater numbers of women.

Over the first 72 hours after delivery, 1,360 puerperal women at all the hospitals in the municipality were approached to invite them to participate in the study. Ten did not accept the invitation, four did not know their full address and two came from violent areas, which made home follow-up impossible. A total of 1,344 mothers were included in the study.

The present study used information from the questionnaires applied at the maternity hospital. The research instruments were drawn up using clear and objective language, with closed answers (yes, no and I don’t know) and were applied in the form of a direct interview by previously trained healthcare professionals.

### Variables

Women within the supplementary health network (those who used health plans) or those who paid the expenses of their own childbirth were considered to be part of the private sector. The deliveries that took place in hospitals connected to the BNHS were classified as public-sector care.

The other characteristics evaluated were: maternal age (<20, ≥20 years); parity (primiparous, multiparous); mother’s educational level (≤8 years,>8 years); mother working outside of the home; mother’s skin color (white, black/brown); family income (<2 minimum monthly salaries, ≥2 minimum monthly salaries); living with the child’s father; number of prenatal consultations (<6 consultations, ≥6 consultations); prenatal care in the private network or prenatal at the same place as childbirth; hospital healthcare provision at low or high complexity level, according to whether a NICU was available and where healthcare was provided for high-risk pregnancies; day of delivery (between Wednesday and Friday or on other days); number of deliveries during the medical shift (<8 childbirths, ≥8 childbirths); birth weight (<2,500 grams, ≥2,500 grams); twinning; and sex of the newborn.

### Statistical analysis

The database was doubly entered by scientific initiation students, within the Statistical Package for the Social Sciences (SPSS) for Windows version 16.0 (Chicago, IL, USA) and was subjected to validation with the help of statistical package Epidata. The analysis was divided into two parts: descriptive and analytical. In the descriptive analysis, the characteristics of the women who gave birth in public and private hospitals were compared. P-values ≤ 5 % were considered significant in calculating the differences between the respective proportions.

Multivariate analysis was carried out using logistic regression in a hierarchical analysis model. In the first level (distal), maternal socioeconomic and demographic variables were entered; in the second level (intermediate), the prenatal healthcare and delivery factors were added; and in the third level (proximal characteristics), the child demographics were added. Initially, all the variables included in the model had a value of p ≤ 0.20. After removing the variables that did not reach statistical significance (p ≤ 0.05) in the first level, the variables of the second level were added. This process was continued until the last group of variables (proximal) had been included. During the process of constructing the final model, the resulting variables of the previous groups were kept even when they lost their significance. With the intent of enabling comparisons among the magnitudes of the effects of each variable on cesarean delivery, between the types of service, the variables that were significant in the public model were maintained for the private model and vice versa.

The analysis was carried out in the same way and with the same variables in both models (public and private), except regarding the following variables: level of hospital care, number of deliveries during the medical shift and twinning. This was because in the sample analyzed, the private sector hospitals were not characterized as high-complexity services, since they did not provide medical care for high-risk pregnant women, they had low volumes of patients (<8 deliveries/day) and they did not have any cases of twins to care for during the period.

### Ethical aspects

This study was approved by the Research Ethics Committee of Feira de Santana State University (protocol 012/2003). Furthermore, all ten participating hospitals only allowed data collection in their respective units upon submission of approval by the Committee of Research Ethics. All women included in the study provided written informed consent.

## Results

### Study population and prevalence of deliveries

A total of 1,344 women were assessed, of whom 75.8 % (1019) gave birth in establishments connected to BNHS. The prevalence of surgical deliveries in the sample was 43.5 % (585/1,344). In the private and public sectors, the occurrence of cesareans deliveries was, respectively, 86.2 % (280/325) and 29.9 % (305/1019).

The characteristics of the sample according to whether the delivery occurred in the public or private service are described in Table 1. Significant differences were observed between the groups. The women who sought medical care through BNHS were younger, had higher parity, lower educational level, less frequently worked outside of the home, had a lower income, presented greater proportions of black and brown skin colors, and not living together with child’s father. Regarding the healthcare characteristics, mothers attended through BNHS had lower numbers of prenatal consultations and gave birth at hospitals providing higher-complexity care and with greater numbers of births per shift. Some of these women had had prenatal care in private clinics. No statistically significant differences were observed between BNHS and the private network regarding the following variables: prenatal care at the place of delivery, birth weight, sex of the child, and day on which procedure took place.

**Table 1:** Comparison of the characteristics of the sample according to whether birth took place in a public or a private hospital

Variables	Hospital category				p-value
	Public		Private		
	N	%	N	%	
Maternal age	1019	100.0	325	100.0	
<20 years	239	23.5	23	7.1	0.000
≥20 years	780	76.5	302	92.9	
Parity	1019	100.0	325	100.0	
Primiparous	482	47.3	189	58.2	0.001
Multiparous	537	52.7	136	41.8	
Maternal educational level	1019	100.0	325	100.0	
≤8 years	485	47.6	23	7.1	0.000
>8 years	534	52.4	302	92.9	
Working outside of the home	1019	100.0	325	100.0	
Yes	227	22.3	198	60.9	0.000
No	792	77.7	127	39.1	
Maternal skin color	1019	100.0	325	100.0	
White	155	15.2	90	27.7	0.000
Black or brown	864	84.8	235	72.3	
Family income	1019	100.0	325	100.0	
<2 minimum monthly salaries	709	69.6	26	8.0	0.000
≥2 minimum monthly salaries	310	30.4	299	92.0	
Living with child’s father	1019	100.0	325	100.0	
Yes	842	82.6	303	93.2	0.000
No	177	17.4	22	6.8	
Number of prenatal consultations	1019	100.0	325	100.0	
<6 consultations	342	26.2	10	3.1	0.000
≥6 consultations	677	73.8	315	96.9	
Prenatal care in private network	972	100.0	325	100.0	
Yes	86	8.8	323	99.4	0.000
No	886	91.2	2	0.6	
Prenatal care at the place of childbirth	972	100.0	325	100.0	
Yes	123	12.7	50	15.4	0.221
No	849	87.3	275	84.6	
Level of hospital care	1019	100.0	325	100.0	
Low complexity	927	91.0	325	100.0	0.000
High complexity	92	9.0	0	0.0	
Day when childbirth occurred	1019	100.0	325	100.0	
Between Wednesday and Friday	483	47.4	146	44.9	0.444
Other days	536	52.6	179	55.1	
Volume of deliveries during the shift	1019	100.0	325	100.0	
<8 deliveries/24 hours	495	48.6	325	100.0	0.000
≥8 deliveries/24 hours	524	51.4	0	0.0	
Birth weight	1019	100.0	325	100.0	
<2500 grams	55	5.4	11	3.4	0.184
≥2500 grams	964	94.6	314	96.6	
Twinning	1019	100.0	325	100.0	
Yes	14	1.4	0	0.0	0.028
No	1005	98.6	325	100.0	
Sex	1019	100.0	325	100.0	
Male	555	54.5	164	50.5	0.225
Female	464	45.5	161	49.5	

### Maternal, child and healthcare factors associated with cesarean delivery according to the type of service

Table 2 presents the prevalences of cesarean deliveries in the public and private service, according to the exposure variables. The logistic regression model showed that the predictive risk factors for cesarean delivery in the public service (Table 3) were: primiparity, twinning, prenatal care provided within the private network and birth in a hospital that provides high-complexity care. On the other hand, the protection factors were the mother’s age below 20 years and a lower number of prenatal consultations. Unlike the public service, the private sector (Table 4) did not present any statistically significant association between the characteristics studied and cesarean delivery.

**Table 2:** Bivariate analysis on the characteristics associated with cesarean delivery in public and private hospitals

Variables	Private			Public		
	Cesarean delivery		PR (95 % CI)	Cesarean delivery		PR (95 % CI)
	Yes n (%)	No n (%)		Yes n (%)	No n (%)	
Maternal age						
<20 years	19 (82.6)	4 (17.4)	0.95 (0.79 - 1.16)	52 (21.8)	187 (78.2)	0.67 (0.52 - 0.87)
≥20 years	261 (86.4)	41 (13.6)		253 (32.4)	527 (67.6)	
Parity						
Primiparous	163 (86.2)	26 (13.8)	1.00 (0.92 - 1.10)	160 (33.2)	322 (66.8)	1.23 (1.02 - 1.48)
Multiparous	117 (86.0)	19 (14.0)		145 (27.0)	392 (73.0)	
Maternal educational level						
≤8 years	19 (82.6)	4 (17.4)	0.95 (0.79 - 1.16)	125 (25.8)	360 (74.2)	0.76 (0.63 - 0.93)
>8 years	261 (86.4)	41 (13.6)		180 (337)	354 (66.3)	
Working outside of the home						
Yes	177 (89.4)	21 (10.6)	1.10 (1.00-1.21)	85 (37.4)	142 (62.6)	1.35 (1.10 - 1.65)
No	103 (81.1)	24 (18.9)		220 (27.8)	572 (72.2)	
Maternal skin color						
White	79 (87.8)	11 (12.2)	1.03 (0.94 - 1.13)	63 (40.6)	92 (59.4)	1.45 (1.17 - 1.81)
Black or brown	201 (85.5)	34 (14.5)		242 (28.0)	622 (72.0)	
Family income						
<2 minimum monthly salaries	20 (76.9)	6 (23.1)	0.88 (0.71 - 1.09)	182 (26.5)	521 (73.5)	0.70 (0.58 - 0.85)
≥2 minimum monthly salaries	260 (87.0)	39 (13.0)		117 (37.7)	193 (62.3)	
Living with the child’s father						
Yes	259 (85.5)	44 (14.5)	0.90 (0.81 - 1.00)	261 (31.0)	581 (69.0)	1.25 (0.95 -1.64)
No	21 (95.5)	1 (4.5)		44 (24.9)	133 (75.1)	
Prenatal care						
<6 consultations	7 (70.0)	3 (30.0)	0.80 (0.54 - 1.22)	87 (25.4)	255 (74.6)	0.79 (0.64 - 0.98)
≥6 consultations	273 (86.7)	42 (13.3)		218 (32.2)	459 (67.8)	
Prenatal care in the private network						
Yes	279 (86.4)	44 (13.6)	1.73 (0.43 - 6.91)	43 (50.0)	43 (50.0)	1.77 (1.40 - 2.24)
No	1 (50.0)	1 (50.0)		250 (28.2)	636 (71.8)	
Prenatal care at the place of childbirth						
Yes	41 (82.0)	9 (18.0)	0.94 (0.82 - 1.08)	47 (38.2)	76 (61.8)	1.32 (1.03 - 1.69)
No	239 (86.9)	36 (13.1)		246 (29.0)	603 (71.0)	
Hospital complexity						
Low	280 (86.2)	45 (13.8)	---------	259 (27.9)	668 (72.1)	0.56 (0.44 - 0.70)
High	------	------		46 (50.0)	46 (50.0)	
Day of delivery						
Between Wednesday and Friday	129 (88.4)	17 (11.6)	1.05 (0.96 - 1.14)	153 (31.7)	330 (68.3)	1.12 (0.93 - 1.35)
Other days	151 (84.4)	28 (15.6)		152 (28.4)	384 (71.6)	
Number of deliveries						
<8 deliveries in 24 hours	280 (86.2)	45 (13.8)	---------	171 (34.5)	324 (65.5)	1.33 (1.12 - 1.63)
≥8 deliveries in 24 hours	------	------		134 (25.6)	390 (74.4)	
Birth weight						
<2500 grams	9 (81.8)	2 (18.2)	0.95 (0.72 - 1.26)	17 (30.9)	38 (69.1)	1.03 (0.69 - 1.55)
≥2500 grams	271 (86.3)	43 (13.7)		288 (29.9)	676 (70.1)	
Twinning						
Yes	------	------	---------	8 (57.1)	6 (42.9)	1.93 (1.22 - 3.07)
No	280 (86.2)	45 (13.8)		297 (29.6)	708 (70.4)	
Sex						
Male	140 (85.4)	24 (14.6)	0.98 (0.90 - 1.07)	167 (30.1)	388 (69.9)	1.01 (0.84 - 1.22)
Female	140 (87.0)	21 (13.0)		138 (29.7)	326 (70.3)

**Table 3:** Factors associated with cesarean delivery in public hospitals according to hierarchical logistic regression

Variables	Model 1- Distal	Model 2 – Intermediate	Model 3 – Proximal
	RR (95 % CI)	RR (95 % CI)	RR (95 % CI)
Lower maternal age (<20 years)	0.68 (0.51 - 0.91)	0.65 (0.48 - 0.87)	0.64 (0.48 - 0.87)
Primiparous mother	1.32 (1.06 - 1.64)	1.35 (1.09 - 1.67)	1.37 (1.10 - 1.69)
Lower maternal educational level (<8 years)	0.93 (0.75 - 1.15)	---------	---------
Mother working outside of the home	1.25 (1.00 - 1.55)	1.20 (0.96 -1.51)	1.21 (0.97 - 1.52)
White mother	1.28 (1.00 - 1.63)	---------	---------
Lower family income (<1 minimum monthly salary)	0.80 (0.65 - 0.99)	0.84 (0.68 - 1.04)	0.83 (0.67 - 1.02)
Mother living with child’s father	1.19 (0.90 - 1.59)	---------	---------
Prenatal care with less than 6 consultations	---------	0.78 (0.62 - 0.98)	0.78 (0.62 - 0.98)
Prenatal care in the private network	---------	1.48 (1.15 - 1.90)	1.44 (1.12 - 1.85)
Prenatal care at the place of childbirth	---------	1.26 (0.96 - 1.66)	---------
Birth in low-complexity hospital	---------	0.60 (0.46 - 0.79)	0.59 (0.46 - 0.77)
Birth in hospital with < 8 deliveries/24 h	---------	1.25 (1.00 - 1.55)	1.21 (0.97 - 1.51)
Twin childbirth	---------	---------	1.77 (1.03 - 3.04)

**Table 4:** Factors associated with cesarean delivery in private hospitals according to hierarchical logistic regression

Variables	Model 1- Distal	Model 2 – Intermediate	Model 3 – Proximal
	RR (95 % CI)	RR (95 % CI)	RR (95 % CI)
Lower maternal age (<20 years)	0.97 (0.80 - 1.18)	---------	---------
Primiparous mother	1.00 (0.92 - 1.09)	---------	---------
Lower maternal educational level (<8 years)	1.01 (0.86 - 1.17)	---------	---------
Mother working outside of the home	1.10 (0.99 - 1.21)	---------	---------
White mother	1.02 (0.93 - 1.12)	---------	---------
Lower family income (<1 minimum monthly salary)	0.91 (0.74 - 1.11)	---------	---------
Mother living with child’s father	0.89 (0.82 - 0.96)	0.89 (0.82 - 1.00)	---------
Prenatal care with less than 6 consultations	---------	0.84 (0.56 - 1.26)	---------
Prenatal care in the private network	---------	1.41 (0.45 - 4.39)	---------
Prenatal care at the place of childbirth	---------	0.96 (0.84 - 1.09)	---------
Birth in low-complexity hospital	---------	---------	---------
Birth in hospital with < 8 deliveries/24 h	---------	---------	---------
Twin childbirth	---------	---------	---------

## Discussion

### Study population and childbirth prevalence

In the present study, the prevalence of cesarean delivery was 43.5 % in the municipality of Feira de Santana in 2004. This percentage was higher than the average for Brazil (41.8 %), for the northeastern region (30.5 %) and for the state of Bahia (25.8 %), and was similar to that of the state capital (43.0 %), according to the Basic Health Indicators for Brazil in 2004 [5]. However, all these rates greatly exceed the WHO recommendation, which was defined almost three decades ago and establishes a maximum limit of 15 % for this procedure [3]. In this regard, it is necessary to point out the need to review this recommended rate, considering recent incorporations of technology, such as artificial insemination, and the increases in twinning and premature births, as well as behavioral changes in the female population, with childbirth at more advanced ages.

There was a large difference regarding the occurrence of cesarean deliveries according to whether the birth took place in a public or a private service. In the municipality examined, the proportion was 29.9 % in the public sector, while in the private sector it was 86.2 %. Other authors have confirmed that there is an evident disparity between the two types of healthcare services, with greater prevalence of cesarean deliveries in the private sector [2, 11, 13, 14].

In the sample studied, there were also great discrepancies in the population characteristics of the women that gave birth in each type of healthcare service. These results are in agreement with those of the National Demography and Health Survey for children and women, carried out in 2006, in which differences in the prevalence of surgical deliveries between the private network (80.8 %) and public services (33.6 %) were observed, as well as greater frequency of surgical deliveries among women with greater educational level and higher economical classification [15].

### Maternal, child and healthcare factors associated to cesarean delivery

Even with a lower prevalence of surgical delivery in the public sector, the results still show a cause for concern. The maternal characteristics that were associated with a cesarean outcome in the public sector were primiparity, twinning and greater maternal age.

The finding of higher cesarean rates among primiparous women is troubling, since this contributes to increased prevalence of surgical deliveries, given that many doctors consider that performing a cesarean delivery previously is indicative of new cesarean [16, 17]. This situation shows that there needs to be a more careful examination of the indications for primary cesareans deliveries, such as in twin pregnancies, because of the controversies that exist in relation to the true benefits of this choice for newborns, for which vaginal delivery seems to be a viable option [18].

Another condition observed in the present study for primary indication of cesareans deliveries, which deserves attention, is greater maternal age. The literature points to greater maternal age as an important factor associated with cesareans deliveries because of a greater chance of comorbidities, which would justify the surgical procedure [10, 13, 19].

The quality of the information and suggestions shared by the healthcare services during prenatal care can also influence the means of birth. In the present study, pregnant women who had their prenatal care in the private sector presented a greater frequency of cesareans deliveries, even when their delivery labor was provided by healthcare services connected to BNHS. This same result was observed in 2006 [13]. It is possible that a previous maternal decision, influenced by medical training and the characteristics of the healthcare services, also contributes towards defining the type of delivery, thereby selecting a cesarean. Moreover, a disconnect between the information from prenatal care and the delivery care can contribute towards the decision of the doctor on duty to implement a cesarean delivery.

Another discussion point is the direct relationship between the number of prenatal consultations and the greater probability of surgical deliveries. Prenatal care is the time when a set of practices and attitudes seeking to promote healthy delivery and birth that preserve women’s autonomy and avoid unnecessary interventions is instigated. Prenatal care is the ideal time to prepare a woman for a vaginal delivery, since the desire, intention and decision for the type of childbirth is defined during this period [20]. However, in the present study, having six or more prenatal consultations was associated with a higher probability of surgical delivery. Other studies corroborate this result, thus demonstrating that a greater number of consultations allows greater negotiation and bonding between the doctor and the patient, which contributes towards a cesarean outcome [13].

Births in maternity hospitals that are classified as providing high-complexity care also contributed towards cesarean deliveries. A greater rate of cesarean deliveries in higher-complexity maternity hospitals is expected, since these hospitals are referral points for obstetric follow-up of high-risk pregnancies, which because of their characteristics require interventionist procedures more frequently. It is important to emphasize that, in the municipality of Feira de Santana, only the public hospitals that were responsible for the greatest volume of childbirths had NICUs.

The absence of environmental variables as determinant factors for the means of birth in the private sector drew attention. It seems that the obstetric healthcare model, the doctor-patient relationship, the maternal desire, and medical factors prevailed over socioeconomic and demographic characteristics. Studies have demonstrated that pregnant women accept or ask for a surgical procedure, electively [20, 21].

For doctors, their experience of a commercialized and interventional biomedical model, the natural length of time over which delivery labor evolves, insecurity when faced with unexpected events and fear of legal liability, as well as the impossibility of having a work agenda with a comfortable schedule, is making obstetricians opt more frequently for interventionist and programmed procedures, thereby interfering in women’s choices to influence them towards a cesarean outcome [22, 23].

Although no association between sociodemographic characteristics and the type of delivery in private health services was observed in the current study, it should be taken into account that high income and education can facilitate women’s access to private health services. Thus, the greater number of cesarean deliveries in private health services may not be due to the particularities of the doctors or patients in these services, but rather to the fact that the women attended to in these services have a uniformly high socioeconomic status, as they are able to pay for the procedure, different than those attended to in the public system.

The disparities found between the proportions of cesareans deliveries in the public and private service bring social inequity in healthcare to the core of the discussion. Women with more privileged socioeconomic characteristics are having differentiated attention, even taking into consideration the risk to the woman’s and child’s health from excessively interventional procedures. On the other hand, women with lower socioeconomic status may have limited access to cesarean delivery, even when there is a medical indication to cesarean delivery due to risk of morbidity and mortality among women and newborns. Other researchers when evaluating birth by cesarean delivery in developing countries have demonstrated this fact [4].

Although a definition of the ideal cesarean rate for society exists, the indication for a cesarean delivery needs to be defined by the clinical conditions of the mother and fetus, which do not differ substantially among the various healthcare services in different regions in the world. The autonomy of women and power to be able to choose the type of birth must also be considered.

### Methodological considerations

Lastly, it is necessary to acknowledge some methodological limitations inherent to this study design. The present design does not allow any affirmation of cause versus effect among the factors studied, even considering that the data analyzed came from a cohort study. Thus the model used is only partially capable of explaining the outcome even considering the data is almost a decade old, particularly because no structural changes have occurred in the health care system in the city in the interim period.

Although the analysis was conducted in the same manner and with the same variables in both models, this does not eliminate the possibility that in other studies, another variable not included in this study could be associated with the outcome. One example of this is gestational age, which was not included in this analysis because the data was collected by direct interviews with the mothers, who were not able to provide an exact number of weeks of gestation. This analysis also did not control for high-risk pregnancy, however this was accounted for by the level of complexity of the hospitals included in the study.

## Conclusions

The present study showed high prevalence of cesarean deliveries, especially in private hospitals. The absence of determinants for cesareans deliveries in the private sector draws attention to the possibility that the variables investigated were masked by unmeasured factors, such as: institutional and health insurance plan interests; desire among doctors and mothers; and academic influence based on a care model with high incorporation of technology.

In the public sector, the population groups with greatest vulnerability were mothers who were primiparae, had twin pregnancies, and mothers who were 20 years or older. These are the usual indications for primary cesareans deliveries, and they often have the implication of future procedures. Such management needs to be reviewed. In intervention measures that aim to reduce the high proportion of cesarean deliveries, the quality of prenatal care and delivery care also need to be evaluated, even in hospitals providing high-complexity services, without excluding the possibility of vaginal delivery.

## Abbreviation

BNHS: Brazilian National Health System
NICU: Neonatal intensive care unit
WHO: World Health Organization.
